# Expectation Modulates the Effect of Deep Brain Stimulation on Motor and Cognitive Function in Tremor-Dominant Parkinson's Disease

**DOI:** 10.1371/journal.pone.0081878

**Published:** 2013-12-02

**Authors:** Ariane Keitel, Stefano Ferrea, Martin Südmeyer, Alfons Schnitzler, Lars Wojtecki

**Affiliations:** 1 Institute of Clinical Neuroscience and Medical Psychology, Medical Faculty, Heinrich-Heine-University, Duesseldorf, Germany; 2 Department of Neurology, University Hospital, Medical Faculty, Heinrich-Heine-University, Duesseldorf, Germany; Hospital General Dr. Manuel Gea González, Mexico

## Abstract

Expectation contributes to placebo and nocebo responses in Parkinson's disease (PD). While there is evidence for expectation-induced modulations of bradykinesia, little is known about the impact of expectation on resting tremor. Subthalamic nucleus (STN) deep brain stimulation (DBS) improves cardinal PD motor symptoms including tremor whereas impairment of verbal fluency (VF) has been observed as a potential side-effect. Here we investigated how expectation modulates the effect of STN-DBS on resting tremor and its interaction with VF. In a within-subject-design, expectation of 24 tremor-dominant PD patients regarding the impact of STN-DBS on motor symptoms was manipulated by verbal suggestions (positive [placebo], negative [nocebo], neutral [control]). Patients participated with (MedON) and without (MedOFF) antiparkinsonian medication. Resting tremor was recorded by accelerometry and bradykinesia of finger tapping and diadochokinesia were assessed by a 3D ultrasound motion detection system. VF was quantified by lexical and semantic tests. In a subgroup of patients, the effect of STN-DBS on tremor was modulated by expectation, i.e. tremor decreased (placebo response) or increased (nocebo response) by at least 10% as compared to the control condition while no significant effect was observed for the overall group. Interestingly, nocebo responders in MedON were additionally characterized by significant impairment in semantic verbal fluency. In contrast, bradykinesia was not affected by expectation. These results indicate that the therapeutic effect of STN-DBS on tremor can be modulated by expectation in a subgroup of patients and suggests that tremor is also among the parkinsonian symptoms responsive to placebo and nocebo interventions. While positive expectations enhanced the effect of STN-DBS by further decreasing the magnitude of tremor, negative expectations counteracted the therapeutic effect and at the same time exacerbated a side-effect often associated with STN-DBS. The present findings underscore the potency of patients' expectation and its relevance for therapeutic outcomes.

## Introduction

In Parkinson's disease (PD), dopamine replacement therapy and deep brain stimulation (DBS) of the subthalamic nucleus (STN) are well established and effective treatments for the cardinal symptoms resting tremor, bradykinesia and rigidity [1]–[3]. Although both treatments generally lead to improvement in motor symptoms, tremor is usually more effectively supressed by STN-DBS as compared to dopamine replacement therapy (for a review see [4]). Moreover, while STN-DBS does not affect overall cognitive function [5], [6], adverse effects of therapeutic STN-DBS have been reported for verbal fluency [7]–[9].

There is considerable evidence that PD is among the disorders in which placebo and nocebo responses play a significant role and can thus contribute to the outcome of treatments. The occurrence of placebo and nocebo responses in PD has been observed in pharmacological placebo-controlled clinical trials [10]–[12] and in studies experimentally investigating the role of expectation as one of the main mechanisms mediating those responses (for reviews see [13], [14]). For example, the administration of placebo drugs which PD patients expect to be a potent antiparkinsonian medication induces a substantial dopamine release in the striatum and alterations in the firing rate of single neurons in the STN associated with improvement in rigidity [15]–[17]. Furthermore, the therapeutic effect of STN-DBS on bradykinesia can be modulated by verbally induced opposite expectations regarding the effect of STN-DBS with improvement following positive expectation and impairment in consequence of negative expectation [18]–[21]. Interestingly, a modulation of verbal fluency has been described in relation to expectation-induced placebo responses in bradykinesia in PD patients treated with STN-DBS [21].

While it has been repeatedly shown that bradykinesia and rigidity are responsive to verbally induced expectation [15], [18]–[21], research regarding its effect on resting tremor in PD patients treated with STN-DBS is scarce. Given evidence of a worsening of resting tremor in PD patients performing cognitive tasks or during mental stress [22], it is of clinical relevance to investigate whether the therapeutic effect of STN-DBS on tremor can also be modulated by patients' expectations. Furthermore, while there is strong evidence for expectation-induced placebo responses, much less is known about nocebo responses in PD which have only been described for bradykinesia in two studies so far [18], [20]. Thus, the primary aim of the present study was to systematically investigate how differing expectations induced by verbal suggestions (positive [placebo], negative [nocebo], neutral [control]) modulate the therapeutic effect of STN-DBS on resting tremor and its interaction with verbal fluency in tremor-dominant PD patients. The secondary aim was to study the effect of expectation regarding STN-DBS on proximal and distal movements.

The present study was part of a transregional and translational research unit investigating the role of conditioning and expectation as underlying mechanisms of placebo and nocebo responses in different physiological systems, pathophysiological conditions and therapeutic interventions, where we set out to examine the effect of expectation on motor and cognitive functions in PD patients treated with STN-DBS.

## Materials and Methods

### Participants

Twenty-four Parkinson's disease patients of the tremor-dominant subtype (19 men and 5 women, mean age: 64.17±1.6 [SEM] years, range: 45–75) with chronic bilateral STN-DBS participated in the study. Patients were recruited from the Movement Disorder Centre of the University Hospital Duesseldorf. In order to rule out a possible cognitive impairment and clinically relevant depressive symptoms all patients were tested with the Mattis Dementia Rating Scale (MDRS [23]) with a cut-off score of ≤130 and filled in the Beck Depression Inventory [24] with a cut-off score for clinically relevant depression of ≥18 before study participation. For patients' characteristics and stimulation parameters see Table S1 and Table S2.

### Experimental Design and Procedure

Using a repeated-measures design, three expectation conditions (positive [placebo], negative [nocebo], neutral [control], see below) were applied in a counterbalanced order and patients were randomly assigned to one of six possible orders. Patients participated twice on two consecutive days, one day on (MedON) and one day off (MedOFF) antiparkinsonian medication, i.e, after withdrawal from any antiparkinsonian medication for at least twelve hours prior to study participation.

The experimental sessions were performed at the Department of Neurology of the University Hospital Duesseldorf. Different expectations (positive, negative, neutral) were induced using verbal suggestions. Prior to each verbal suggestion, STN-DBS was switched off for ten minutes. Before STN-DBS was turned on again patients' expectations regarding the effect of STN-DBS on motor symptoms were verbally manipulated by an experienced movement disorders physician (L.W., M.S., S.F.). In the positive expectation condition, patients were informed that parameter settings of the upcoming stimulation would be adjusted in order to strongly improve tremor and motor function in general (placebo condition). That is, in the aforementioned condition patients were told the following: ‘The upcoming stimulation will be turned on with parameter settings which will effectively improve tremor and motor function and thus will considerably improve your current motor state’. In contrast, in the negative expectation condition patients were told that the subsequent stimulation would strongly worsen tremor and motor function (nocebo condition). In the neutral expectation condition, patients were informed that the upcoming stimulation would not have any impact on tremor and motor function (control condition). Thus, the neutral expectation condition in which no specific expectation was induced served as a control condition considered to reflect the genuine STN-DBS effect. The text used for verbal suggestions was standardized and the physician who induced expectations was held constant for each patient. After expectations were verbally induced, STN-DBS was turned on (Stim ON) according to the patient's individual therapeutic settings in each condition. This means that stimulation parameters (intensity, frequency and pulse width) were identical in all three conditions, a fact patients were blinded to. In between the conditions, the stimulator was switched off for ten minutes. STN-DBS usually improves symptoms such as rigidity and tremor in less than a minute and improvement in bradykinesia is gradually achieved within a couple of minutes [25]. Consequently, in each condition assessment of dependent variables was undertaken after STN-DBS had been turned on for 15 minutes. The experimental session lasted about 120 minutes per day. For more details of the procedure see Keitel et al. [21].

#### Expectation Rating

Directly after expectations were verbally induced, patients rated to what degree they expected improvement, impairment or no change of their current motor state on a numeric rating scale. The numeric rating scale ranged from +5 indicating expectation of strong improvement to −5 indicating expectation of strong impairment of motor function while 0 represented expectation of no change of motor function.

#### Motor Function: Resting Tremor, Distal and Proximal Movements

Resting tremor was objectively determined by means of an accelerometer whose signal was recorded using an analogue channel of a 3D ultrasound motion detection system (CMS 70P v 5, Zebris, Isny, Germany). In order to assess resting tremor, the accelerometer was attached to the patient's hand of the clinically more affected body side. Tremor was then recorded during 30 seconds of rest in each condition. For recording, patients were seated in a chair with bilateral armrests, placed the hand of the clinically more affected side as comfortable as possible on the armrest and were asked to avoid any voluntary movements.

Moreover, the ultrasound motion detection system was used to assess performance in proximal (diadochokinesia) and distal (finger tapping) movements. For the assessment of diadochokinesia, patients were asked to rotate using a wooden bar with two 3D markers (ultrasound transmitters) attached to each end. To record finger tapping, two ultrasound transmitters were attached to the patients' hand; one to the lateral side of the index finger tip and one to the thumbnail. Patients performed three trials of 10 seconds of finger tapping as well as of diadochokinesia using the hand/arm of the clinically more affected side in each condition. Between the trials, patients paused for a period of 30 seconds. Details regarding the assessment of diadochokinesia and finger tapping can be found elsewhere [21].

#### Cognitive Function: Verbal Fluency

Verbal fluency was assessed using four different tests: a formal lexical test, a semantic category test, a formal lexical category change test and a semantic category change test. In each test, patients were asked to produce as many words as possible within a time period of one minute. In the formal lexical test patients were instructed to produce words beginning with a specific letter (e.g. ‘S’). In the semantic category test they had to name words of a certain semantic category (e.g. ‘animals’). In the formal lexical category change test, patients were asked to switch between two different letters (e.g. a word beginning with the letter ‘G’ followed by a word beginning with the letter ‘R’). In the semantic category change test patients had to alternate between two semantic categories (e.g. ‘clothes’ and ‘flowers’).

As dependent variables were assessed six times throughout the experimental sessions (three conditions in MedON and in MedOFF, respectively), six parallel test versions of each verbal fluency test were employed in a randomized order to avoid learning effects.

#### Questionnaires

To identify potential mediators of placebo and nocebo responses, patients' state and trait anxiety were assessed using the STAI-S and STAI-T questionnaire [26]. Moreover, patients were asked to fill in a questionnaire on beliefs about medicines [27] which assesses general and specific views about medicines.

### Ethics

According to the cognitive screening (MDRS-scores, see Materials and Methods as well as Table S1) none of the patients who participated in the present study was cognitively impaired and had thus no compromised capacity to consent. All patients gave informed, written consent themselves. The study was approved by the local ethics committee of the Medical Faculty, Heinrich-Heine-University (study no. 3403), Duesseldorf, Germany and was in accordance with the standards of the declaration of Helsinki guidelines.

### Data Analysis and Statistics

Data of tremor as well as of distal (i.e. finger tapping) and proximal (i.e. diadochokinesia) movements were stored on the recording PC's hard disk and analyzed offline. Each data set was inspected offline for artifacts. Epochs containing artifacts were excluded from further analysis. Data were analyzed using custom-made MATLAB™ 7.1 (The Mathworks Inc., Natick, MA, USA) scripts. Tremor was analyzed regarding power at tremor frequency. For each patient, the tremor frequency was determined when STN-DBS was switched off and when patients were off antiparkinsonian medication and power at tremor frequency was assessed. Input to spectral analysis was the signal of the accelerometer. Spectral power at individual tremor frequency ±1 Hz was computed using Welch's method with half-overlapping segments. The segment length equaled twice the sampling rate, i.e. frequency resolution was 0.5 Hz. Additionally, using an exploratory approach, tremor data were further explored with respect to placebo/nocebo responders. Therefore, a placebo/nocebo response was defined as an improvement (placebo) or worsening (nocebo) in resting tremor of at least 10% compared to the control condition (i.e. patients' individual therapeutic STN-DBS with neutral expectation reflecting the actual STN-DBS effect).

Finger tapping was analyzed with respect to mean frequency and additionally regarding the product of mean amplitude and mean frequency. Therefore, the Euclidian distance between the two ultrasound transmitters attached to index finger and thumb was calculated and noise was reduced using Savitzky-Golay filtering (order: 5, frame size: 41). A tap was defined as a local distance minimum and tap amplitudes were determined by the detection of local maxima. The Matlab function ‘findpeaks’was applied to the sign-inverted signal in order to detect local minima. A local minimum was considered to represent a touch of thumb and index finger if it was smaller than an individually adapted threshold. The tapping frequency was defined as the mean number of taps per second. For detection of local maxima the function ‘findpeaks’ was applied to the original signal. A local maximum was required to exceed 0.1 times the signal's standard deviation. Distance traces were checked visually to ensure that individual taps and tap amplitudes were identified correctly.

Diadochokinesia was analyzed with respect to mean angular speed which was calculated as follows: Subtraction of ultrasound transmitter coordinates yielded a vector in 3-dimensional space that represented the pointing direction of the bar at each point in time. Ideally, this vector moves in one plane only. In practice, however, there is usually a plane which contains most but not all of the movements. This plane was estimated by singular value decomposition. Afterwards, we projected the pointing direction vectors onto this plane and calculated the angle with the second singular vector to obtain angular motion. Using a Savitzky-Golay filter (order: 10, frame size: 100), angular motion was smoothed. Angular velocity was computed by calculation of the first derivative of angular motion and angular speed was defined as the absolute of angular velocity. In each condition, only the trial with the best performance in finger tapping and diadochokinesia, respectively, was used for further analysis.

In order to analyze verbal fluency, for each patient the correct number of words was summed up for each subtest in each condition. Then the mean number of words was computed across all patients and compared between conditions.

Prior to all statistical analyses, univariate normal distribution was tested using Kolmogoroff-Smirnov goodness-of-fit test for each variable. Repeated measures analyses of variance (ANOVA) with condition (placebo vs. control vs. nocebo) as repeated measures factor were computed for MedON and MedOFF. Paired *t*-tests were utilized for post-hoc analyses. In case of violation of sphericity, Greenhouse-Geisser corrections were applied. The non-parametric Friedman test was used instead of ANOVA in case of violation of normal distribution, which was the case for tremor data. When multiple comparisons were performed, Bonferroni correction was applied. Comparison of placebo/nocebo responders vs. non-responders was carried out using the non-parametric Mann-Whitney U test. Statistical data analysis was performed using PASW statistics version 18 (SPSS, Chicago, IL). Regarding tremor data, four outliers were excluded prior to further statistical analysis as the values deviated from the respective group mean by more than three standard deviations.

## Results

In brief, expectation did not significantly affect resting tremor on group level but modulated the effect of STN-DBS on resting tremor in a subgroup of patients. Furthermore, verbal fluency was adversely affected in patients showing a nocebo response in resting tremor. On the other hand, bradykinesia of proximal and distal movements was not significantly modulated by expectation. Descriptive data of the results are presented in Table 1.

**Table 1:** Descriptive data of the outcome measures: Mean and standard error of power at tremor frequency, mean angular speed of diadochokinesia, frequency as well as frequency x amplitude of finger tapping and verbal fluency tests.

		MedOFF			MedON	
	Placebo	Control	Nocebo	Placebo	Control	Nocebo
Power at Tremor Frequency (a.u.)	1.47±0.40	1.88±0.74	1.60±0.51	1.03±0.20	1.12±0.22	1.39±0.38
Mean Angular Speed of Diadochokinesia (degree/s)^#^	432.31±33.70	436.38±34.99	425.96±39.09	428.90±28.95	421.47±29.38	441.82±27.76
Frequency of Finger Tapping (tap/s)^#^	2.26±0.13	2.25±0.10	2.32±0.13	2.39±0.16	2.44±0.16	2.41±0.18
Frequency x Amplitude of Finger Tapping^#^	179.00±17.70	182.43±20.98	179.70±17.53	186.60±19.80	187.25±20.42	178.71±21.01
Formal Lexical (no. of words)	9.04±0.89	8.13±0.98	8.39±0.86	8.74±0.98	7.61±0.68	7.26±0.81
Semantic Category (no. of words)	13.87±0.98	13.48±1.47	13.91±1.01	13.48±1.02	13.26±0.86	12.78±0.74
Formal Lexical Category Change (no. of words)	3.30±0.40	3.56±0.48	3.13±0.46	3.57±0.46	3.57±0.30	3.65±0.38
Semantic Category Change (no. of words)	5.61±0.59	5.04±0.54	5.39±0.38	5.78±0.59	6.00±0.62	4.70±0.35

MedOFF =  off antiparkinsonian medication; MedON =  on antiparkinsonian medication; # refers to the clinically more affected side; a.u. =  arbitrary units.

### Effect of Expectation on Tremor

**Figure 1:**
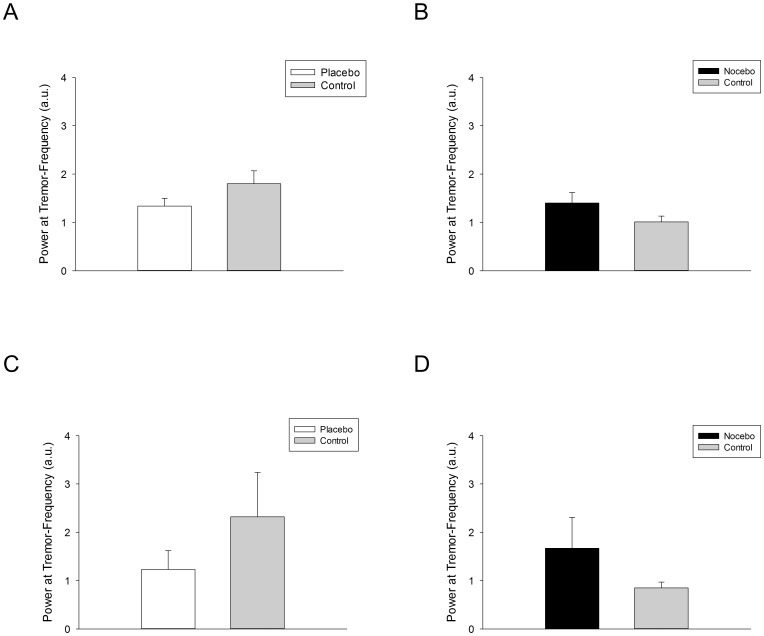
Impact of expectation on resting tremor. Power at tremor frequency (mean and standard error of the mean) in tremor-dominant Parkinson's disease patients treated with deep brain stimulation (DBS) of the subthalamic nucleus (STN). Upper row: Power of tremor in placebo responders in the placebo (open bars) and control condition ([grey bars] n = 8, Fig. 1A) and in nocebo responders in the nocebo (black bars) and control condition (n = 5; Fig. 1B) *on* antiparkinsonian medication. Lower row: Power of tremor in placebo responders (n = 7, Fig. 1C) in the placebo and control condition and in nocebo responders in the nocebo and control condition (n = 2, Fig. 1D) *off* antiparkinsonian medication.

In an exploratory approach, tremor data were inspected individually for the analysis of responders and non-responders. According to the prespecified criterion (i.e. improvement [placebo] or worsening [nocebo] in resting tremor of at least 10% compared to the control condition), eight out of twenty patients showed a placebo response with a mean tremor reduction of −22.84±5.20% (see Fig. 1A) and five patients displayed a nocebo response with a mean tremor increase of 39.00±13.80% (see Fig. 1B) in MedON. In MedOFF, seven patients were characterized by a placebo response with a mean tremor reduction of −38.30±6.77% (see Fig. 1C) and two patients showed a nocebo response with a mean tremor increase of 95.03±72.41% (see Fig. 1D). Yet on group level, expectation did not have a significant effect on resting tremor in MedON and MedOFF (all *p*>0.59).

Moreover, responders and non-responders did not differ significantly with respect to disease-associated variables (disease duration, intake of levodopa equivalent units, duration of chronic bilateral STN-DBS), psychological variables (trait and state anxiety, beliefs about medicine) and expectation rating (placebo responders vs. placebo non-responders: all *p*>0.14; nocebo responders vs. nocebo non-responders: all *p*>0.10).

### Effect of Expectation on Verbal Fluency

**Figure 2:**
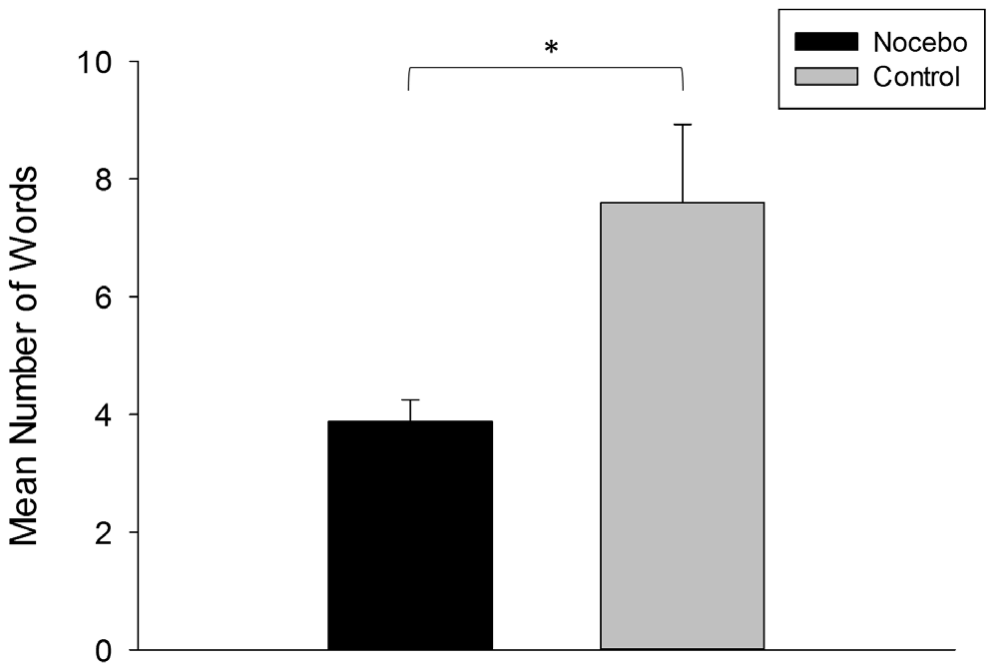
Impact of expectation on verbal fluency in nocebo responders. Performance in verbal fluency of tremor-dominant Parkinson's disease patients showing a nocebo response in tremor: Number of words (mean and standard error of the mean) produced in the semantic category change test in the nocebo condition (black bars) and the control condition (grey bars) in 5 patients on deep brain stimulation of the subthalamic nucleus and on antiparkinsonian medication. * *p*<0.05.

To assess the impact of expectation regarding STN-DBS on verbal fluency, we tested whether it was affected in the subgroups of patients showing a placebo or nocebo response in resting tremor. Therefore, for placebo responders, the mean number of words was compared between the placebo and control condition, separately for the four subtests. For nocebo responders, this comparison was undertaken for the nocebo and control condition. Due to the small sample size of responders, these analyses were performed using the non-parametric Wilcoxon signed-rank test. These analyses revealed that verbal fluency in the semantic category change test was reduced in nocebo responders, i.e., they produced significantly fewer words in the nocebo compared to the control condition (*p*<0.05; see also Fig. 2) whereas no significant effect of expectation was observed for any other verbal fluency subtest (all *p*>0.19). In contrast, no significant effect of expectation on verbal fluency was observed for placebo responders (all *p*>0.28). Moreover, on group level, the three conditions did not differ significantly regarding lexical and semantic verbal fluency in MedOFF and MedON (all *p*>0.12).

### Effect of Expectation on Bradykinesia

Expectation did not significantly affect bradykinesia of distal and proximal movements in MedOFF and MedON, respectively (all *p*>0.39).

### Expectation Rating regarding the Effect of STN-DBS on Motor Function

**Figure 3:**
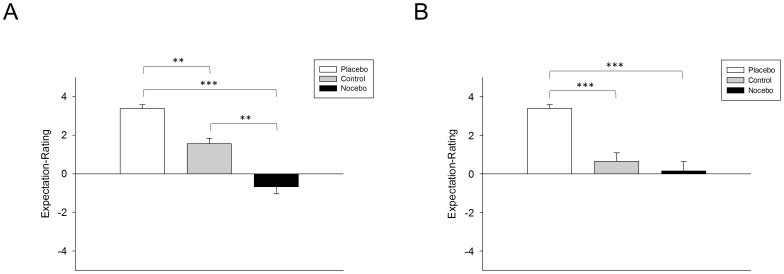
Expectation Rating. Mean and standard error of the mean for the expectation rating under the three conditions (placebo, nocebo, control) when the same Parkinson's disease patients were *on* (n = 24 [Fig. 3A]) and *off* antiparkinsonian medication (n = 24 [Fig. 3B]). On a numeric rating scale patients' expectations regarding the effect of deep brain stimulation of the subthalamic nucleus on motor symptoms were assessed. +5 indicates expectation of strong improvement, −5 indicates expectation of strong impairment while 0 represents expectation of no change of motor function. ** *p*<0.01; *** *p*<0.001

Patients' expectations regarding the effect of STN-DBS on motor function differed significantly in MedON (*F*_(2, 46)_ = 30.87, *p*<0.001) and MedOFF (*F*_(2, 46)_ = 21.30, *p*<0.001), respectively. Post-hoc pairwise comparisons using paired *t*-tests revealed a significant difference between all conditions in MedON (all *p*<0.01; see Fig. 3A). In MedOFF, expectations between the placebo and nocebo condition as well as between the placebo and control condition differed significantly (p<0.001) whereas no significant difference was observed for the comparison between the nocebo and control condition (*t*_(23)_ = −0.90, *p* = 0.38; see Fig. 3B).

## Discussion

The main findings of the present study are that the therapeutic effect of STN-DBS on resting tremor was modulated by verbally induced expectation in a subgroup of PD patients and that negative expectation regarding the STN-DBS effect on motor function also adversely affected verbal fluency in patients showing a nocebo response in resting tremor.

### Effect of Expectation on Tremor

A nocebo increase of tremor subsequently to negative verbal suggestions was observed in some patients when they were *on* and in others when they were *off* antiparkinsonian medication, indicating that the effect of a dopaminergic treatment as well as STN-DBS can be undermined by negative expectations in a subgroup of patients. Given the known phenomenon that tremor often worsens in PD patients experiencing mental stress or performing cognitive tasks [22], the observed increase of tremor in the nocebo condition suggests that expectation of symptom worsening is apparently also a factor which can contribute to tremor aggravation. Regarding expectation-induced placebo responses, subsets of patients *on* as well as *off* antiparkinsonian medication were characterized by a reduction in resting tremor suggesting that expectation of benefit can increase the therapeutic effect of STN-DBS on tremor. Thus, these findings provide evidence that the therapeutic effect of STN-DBS on resting tremor can be modulated by patients' positive and negative expectations and indicate that tremor is also among the parkinsonian symptoms responsive to placebo and nocebo interventions. This view is supported by a study which evaluated the placebo arm of a randomized placebo-controlled pharmacological trial in PD patients assessing the response of motor symptoms to placebo medication. In essence, cardinal motor symptoms such as bradykinesia, rigidity and tremor responded to placebo treatment. Yet tremor was the symptom where the magnitude and occurrence of placebo responses was lowest [10]. Hence, it seems generally possible to modulate tremor by placebo and nocebo treatments although compared to other parkinsonian symptoms such as bradykinesia and rigidity it appears to be the symptom least responsive to those interventions. Interestingly, concordance in patients who showed a placebo as well as a nocebo response was rather low indicating that being responsive to placebo interventions is not necessarily accompanied by proneness to respond to nocebo treatments.

On group level, expectation did not significantly affect resting tremor in the patients of the present study. This finding is in agreement with a study by Mercado et al. [19] who did not observe a modulation of tremor using a different paradigm to manipulate patients' expectation regarding STN-DBS. However, given the relatively small number of placebo and nocebo responders in the present study, the lack of statistical significance on group level is not surprising. In general, the occurrence and extent of placebo and nocebo responses vary considerably across individuals and studies [28]. Thus, the identification of potential psychological, neuroendocrine and genetic factors that might play a role in mediating responsiveness and responses, respectively, is a matter of current debate and investigation in placebo and nocebo research (for a review see [14]). In an attempt to identify factors potentially mediating placebo and nocebo responses in PD, responders and non-responders were compared regarding disease-associated variables, psychological variables and expectation ratings but did not differ significantly with respect to those factors. Consequently, this may indicate involvement of other factors that are related to placebo and nocebo responses in PD which were not assessed in the present study and need to be elucidated in future studies. Moreover, as the subgroup of responders was considerably small, statistical power might not have been sufficient in order to detect significant differences between responders and non-responders.

Another possible explanation for the absence of an effect of negative verbal suggestions on resting tremor in the overall group of the present study might be related to the fact that it is obviously more difficult to induce negative expectations regarding STN-DBS. This interpretation is corroborated by the patients'expectation ratings (see Fig. 3) indicating that on average patients did not expect strong impairment of motor symptoms by STN-DBS in the nocebo condition. Generally, the majority of patients had long-standing beneficial experience with effective suppression of tremor by STN-DBS whereas they usually had not experienced worsening of tremor induced by this treatment and consequently did not establish strong expectations of impairment in the nocebo condition. Indeed, the importance of prior experience with effectiveness or ineffectiveness of a treatment in order to form pronounced expectations regarding its (in-)efficacy, thus providing the basis for the occurrence of placebo and nocebo responses, is also pointed out by studies on placebo analgesia and hyperalgesia [29], [30]. Furthermore, the absence of an expectation-induced effect on resting tremor in the placebo condition on group level might be explained by a floor-effect: As resting tremor was assessed when STN-DBS was switched on, power at tremor frequency was rather low in most patients and barely detectable in some patients in the control condition. Thus, positive expectation could not further substantially decrease resting tremor in the placebo condition. In future studies it would be of interest to assess the impact of expectation on tremor when STN-DBS is - unbeknownst to the patients - switched off to avoid potential floor effects.

There is one limitation regarding the assessment of resting tremor that we would like to address. Given that the amplitude of resting tremor in Parkinson's disease can show considerable variability over time, a longer period of tremor recording would have been useful. However, for practicability reasons - mainly to keep time and effort for the patients on a reasonable level - tremor was only measured for 30 seconds keeping in mind that the study lasted approximately 120 minutes each on two consecutive days. Although we do not have any reason to assume that spontaneous fluctuations in the amplitude of resting tremor varied substantially between conditions, a measurement for a longer period should be considered in future studies to control for potential variability in tremor that might occur over time.

### Effect of Expectation on Verbal Fluency

Those patients who showed an aggravation of resting tremor in the nocebo condition were also characterized by impairment in semantic verbal fluency. Thus, negative expectation regarding the effect of STN-DBS on motor function did not only modulate the magnitude of resting tremor but additionally had an adverse effect on a cognitive function often affected in PD patients treated with STN-DBS [6]–[9]. This suggests an expectation-induced generalization of a nocebo response manifesting on motor as well as on cognitive functions. Yet this finding has to be substantiated in future studies using larger sample sizes in order to increase the likelihood of a greater number of potential responders. In contrast to patients in the subgroup who showed a nocebo response in tremor, no significant effect of expectation on verbal fluency was observed on group level.

In a previous study using the same paradigm as employed in the present study [21], hypokinetic-rigid PD patients who showed a placebo response in bradykinesia were also characterized by a tendency for impairment in lexical verbal fluency. Thus, in contrast to the present study, expectation of beneficial STN-DBS modulated motor and cognitive functions in opposite directions. The discrepant results of an expectation-induced modulation of the STN-DBS effect on motor and cognitive functions in tremor-dominant and hypokinetic-rigid PD patients, indicates that these two PD subtypes do not only differ regarding the clinical phenotype and related underlying pathophysiological patterns such as neuronal oscillations [31], [32], neuroimaging patterns of dopaminergic degeneration [33] and cortical Lewy bodies [34], but diverge also with respect to expectation-induced placebo and nocebo responses and their interaction with verbal fluency.

### Effect of Expectation on Bradykinesia

Expectation did not affect bradykinesia of distal or proximal movements in the patients of the present study. This result differs from the findings of previous studies where a modulation of bradykinesia by expectation was reported [18]–[21]; yet in those studies either exclusively hypokinetic-rigid or a mixture of hypokinetic-rigid and tremor-dominant PD patients were analyzed suggesting that placebo and nocebo responses mainly manifest on symptoms of predominant relevance to the patients, that is, tremor in the patients of the present study. Moreover, although expectations regarding the effect of STN-DBS were not exclusively induced with respect to tremor but also regarding motor symptoms in general, patients may have specifically focused their expectation on tremor rather than on other symptoms such as bradykinesia.

## Conclusion

Taken together, the results of the present study provide evidence that the therapeutic effect of STN-DBS on resting tremor can be modulated by expectation in a subgroup of patients. Moreover, the present findings indicate that tremor is among the parkinsonian symptoms responsive to placebo and nocebo interventions - although less so than other cardinal symptoms. While positive expectations enhanced the effect of STN-DBS by further decreasing the magnitude of resting tremor, negative expectations did not only counteract the therapeutic effect of STN-DBS by increasing the amplitude of tremor, but additionally exacerbated impairment in verbal fluency, a side-effect often associated with therapeutic STN-DBS. This suggests that – at least in a subgroup of patients - negative expectations can undermine the therapeutic effect even of very efficacious treatments such as STN-DBS while at the same time exacerbating side-effects. However, given the relatively small size of responders and the exploratory descriptive approach, future studies are needed to substantiate the findings of the present study and to elucidate the prerequisites and patient-associated factors which contribute to responsiveness to placebo and nocebo interventions in PD. Nevertheless, the present results underscore the potency of patients' expectation and thus its relevance for therapeutic outcomes and should consequently be considered in the context of patient-physician interaction.

## Supporting Information

Table S1 Patient characteristics including sex, age, MDRS- and BDI-scores, disease duration, clinically more affected side, daily antiparkinsonian medication, months since implantation of DBS-electrodes and MDS-UPDRS-scores.
(DOC)

Table S2 Stimulation parameters used for chronic bilateral deep brain stimulation of the subthalamic nucleus.
(DOC)
