# Supplementary material for: Expectation Modulates the Effect of Deep Brain Stimulation on Motor and Cognitive Function in Tremor-Dominant Parkinson's Disease
Source: PLoS One. 2013 Dec 2;8(12):e81878. doi: 10.1371/journal.pone.0081878 (PMC3846869; doi:10.1371/journal.pone.0081878)
Supplement: Table S1 — Patient characteristics including sex, age, MDRS- and BDI-scores, disease duration, clinically more affected side, daily antiparkinsonian medication, months since implantation of DBS-electrodes and MDS-UPDRS-scores. (DOC) [file pone.0081878.s001.doc]

**Table S1.** Patient characteristics including sex, age, MDRS- and BDI-scores, disease duration, clinically more affected side, daily antiparkinsonian medication, months since implantation of DBS-electrodes and MDS-UPDRS-scores.

| **Patient** | **Sex** | **Age (years)** | **MDRS** | **BDI** | **Disease Duration (years)** | **Clinically More Affected Side** | **PD Medication (mg/day)** | **Months since Implantation of DBS-electrodes** | **MDS-UPDRS OFF_MED_/ OFF_STIM_** | **MDS-UPDRS OFF_MED_/ ON_STIM_** | **MDS-UPDRS ON_MED_/ ON_STIM_** |
| --- | --- | --- | --- | --- | --- | --- | --- | --- | --- | --- | --- |
| 1 | m | 69 | 135 | 0 | 4 | left | 1 Rasagiline, 0.54 Pramipexole | 12 | 38 | 19 | 8 |
| 2 | m | 72 | 130 | 7 | 18 | right | 250 L-Dopa, 250 Amantadine, 4 Ropinirole prolonged release | 15 | 69 | 40 | 35 |
| 3 | m | 45 | 144 | 13 | 8 | right | 6 Ropinirole prolongd release, 575 L-Dopa, 50 Amantadine | 48 | 70 | 37 | 33 |
| 4 | m | 60 | 144 | 6 | 7 | left | 300 L-Dopa, 1 Rasagaline | 12 | 44 | 15 | 13 |
| 5 | f | 75 | 140 | 5 | 3 | right | 100 L-Dopa | 3 | 45 | 36 | 35 |
| 6 | m | 66 | 144 | 15 | 17 | right | 100 L-Dopa | 58 | 48 | 21 | 16 |
| 7 | m | 58 | 137 | 17 | 12 | right | 300 L-Dopa, 8 Ropinirole, 150 Amantadine | 99 | 60 | 19 | 10 |
| 8 | m | 74 | 130 | 15 | 12 | right | 550 L-Dopa, 600 Entacapone, 1 Rasagiline | 35 | 55 | 36 | 33 |
| 9 | m | 71 | 130 | 17 | 10 | right | 300 L-Dopa, 600 Entacapone, 1 Rasagaline | 4 | 76 | 40 | 32 |
| 10 | f | 72 | 138 | 4 | 30+ | left | 100 Amantadine, 1 Rasagiline | 108 | 37 | 27 | 27 |
| 11 | f | 67 | 143 | 7 | 12 | left | 1 Rasagaline 100 L-Dopa, 200 Entacapone, 1.4 Pramipexole | 105 | 59 | 24 | 20 |
| 12 | m | 65 | 138 | 9 | 4 | right | 600 L-Dopa, 800 Entacapone, 1 Rasagaline | 4 | 54 | 22 | 20 |
| 13 | m | 50 | 144 | 11 | 19 | left | 1 Rasagaline, 12 Ropinirole prolonged release | 12 | 37 | 20 | 18 |
| 14 | m | 57 | 144 | 4 | 7 | left | 200 L-Dopa, 100 Amantidine, 1 Rasagiline | 3 | 56 | 38 | 34 |
| 15 | f | 58 | 137 | 14 | 19 | right | 10 Ropinirole prolonged release, 1 Rasagiline | 27 | 42 | 25 | 19 |
| 16 | f | 66 | 142 | 3 | 11 | right | 650 L-Dopa, 1200 Entacapone | 4 | 34 | 26 | 25 |
| 17 | m | 60 | 140 | 10 | 8 | left | 50 L-Dopa, 8 Ropinirole prolonged release | 36 | 37 | 18 | 16 |
| 18 | m | 67 | 130 | 11 | 17 | right | 200 L-Dopa, 800 Entacapone, 4 Rotigotine transdermal patch | 15 | 58 | 37 | 25 |
| 19 | m | 69 | 132 | 7 | 5 | left | 150 L-Dopa, 200, 0.35 Pramipexole | 14 | 56 | 32 | 29 |
| 20 | f | 75 | 132 | 17 | 8 | left | 700 L-Dopa, 1200 Entacapone | 4 | 55 | 48 | 22 |
| 21 | m | 75 | 130 | 17 | 8 | right | 200 L-Dopa | 9 | 48 | 31 | 20 |
| 22 | m | 69 | 139 | 6 | 13 | right | 400 L-Dopa | 5 | 36 | 29 | 24 |
| 23 | m | 64 | 144 | 4 | 14 | left | 1 Rasagaline, 6 Ropinirole prolonged release | 27 | 59 | 19 | 8 |
| 24 | m | 65 | 142 | 15 | 12 | right | 100 L-Dopa, 100 Amantadine | 12 | 22 | 10 | 7 |

MDRS = Mattis Dementia Rating Scale; BDI = Beck Depression Inventory; PD = Parkinson’s Disease; DBS = Deep Brain Stimulation; MDS-UPDRS = Movement Disorder Society-sponsored revision of the Unified Parkinson’s Disease Rating Scale; OFF_MED_ = off antiparkinsonian medication; ON_Med_ = on antiparkinsonian medication; OFF_Stim_ = Deep brain stimulation switched off; ON_Stim_ = Deep brain stimulation switched on; m = male; f = female.
