# Supplementary material for: Expectation Modulates the Effect of Deep Brain Stimulation on Motor and Cognitive Function in Tremor-Dominant Parkinson's Disease
Source: PLoS One. 2013 Dec 2;8(12):e81878. doi: 10.1371/journal.pone.0081878 (PMC3846869; doi:10.1371/journal.pone.0081878)
Supplement: Table S2 — Stimulation parameters used for chronic bilateral deep brain stimulation of the subthalamic nucleus. (DOC) [file pone.0081878.s002.doc]

**Table S2**. Stimulation parameters used for chronic bilateral deep brain stimulation of the subthalamic nucleus.

| **Patient** | **Amplitude (V,** if not stated otherwise**)** | | **Pulse Width (μs)** | | **Frequency (Hz)** | | **Contact** | |
| --- | --- | --- | --- | --- | --- | --- | --- | --- |
|  |
|  | left | right | left | right | left | right | left | right |
| 1 | 2.3 | 2.9 | 60 | 60 | 130 | 130 | G+1- | G+9- |
| 2 | 2.3 | 2.6 | 60 | 60 | 130 | 130 | G+10- | G+1- |
| 3 | 2.4 | 1.7 | 60 | 90 | 130 | 130 | G+ 1-,2-,3- | G+6- |
| 4 | 3.0 | 2.9 | 90 | 90 | 140 | 140 | G+1- | G+10- |
| 5 | 2.4 | 0 | 60 | 60 | 130 | 130 | G+2- | G+10- |
| 6 | 2.8 | 2.6 | 60 | 60 | 130 | 130 | G+1- | G+5- |
| 7 | 5.2 | 4.3 | 60 | 60 | 135 | 135 | G+4-,5- | G+0- |
| 8 | 4.1 | 3.5 | 60 | 60 | 130 | 130 | G+0- | G+5- |
| 9 | 3.9 | 2.3 | 60 | 60 | 130 | 130 | G+1- | G+9- |
| 10 | 2.8 | 2.8 | 60 | 60 | 180 | 180 | G+1- | G+5- |
| 11 | 2.3 | 2.4 | 90 | 60 | 125 | 125 | G+3- | G+6-,5-,4- |
| 12 | 3.0 mA | 3.0 mA | 65 | 65 | 130 | 130 | G+2- | G+3- |
| 13 | 3.1 | 2.9 | 60 | 60 | 130 | 130 | G+0 | G+8- |
| 14 | 1.4 mA | 1.2 mA | 65 | 65 | 130 | 130 | G+3- | G+2- |
| 15 | 2.6 | 4.2 | 60 | 60 | 130 | 130 | G+3- | G+10-,11- |
| 16 | 2.0 mA | 2.0 mA | 90 | 90 | 130 | 130 | G+2- | G+2- |
| 17 | 4.4 | 3.2 | 60 | 60 | 150 | 150 | G+0- | G+4- |
| 18 | 2.4 mA | 2.4 mA | 60 | 60 | 130 | 130 | G+1- | G+10- |
| 19 | 3.5 | 4.0 | 60 | 60 | 150 | 150 | G+1- | G+9- |
| 20 | 1.7 | 1.7 | 60 | 60 | 130 | 130 | G+2- | G+10-2 |
| 21 | 3.6 | 1.7 | 90 | 60 | 130 | 130 | G+0 | G+8- |
| 22 | 2.5 | 2.2 | 60 | 60 | 130 | 130 | C+9- | C+1- |
| 23 | 2.6 | 2.6 | 60 | 60 | 130 | 130 | G+1- | G+10- |
| 24 | 0.7 mA | 1.3 mA | 62 | 62 | 130 | 130 | G+2- | G+2- |

V = Volt; mA = Milliampère; μs = Microsecond; Hz = Hertz; Monopolar with impulse generator positive, if not stated otherwise; contact 0 the lowermost contact on each hemisphere.
